# Research on Time Constant Test of Thermocouples Based on QNN-PID Controller

**DOI:** 10.3390/s25123819

**Published:** 2025-06-19

**Authors:** Chenyang Xu, Xiaojian Hao, Pan Pei, Tong Wei, Shenxiang Feng

**Affiliations:** 1 State Key Laboratory of Extreme Environment Optoelectronic Dynamic Measurement Technology and Instrument, North University of China, Taiyuan 030051, China; s202206123@st.nuc.edu.cn (C.X.); b20210622@st.nuc.edu.cn (P.P.); sz202206038@st.nuc.edu.cn (T.W.); b20240602@st.nuc.edu.cn (S.F.); 2 School of Instrumentation and Electronics, North University of China, Taiyuan 030051, China

**Keywords:** thermocouple, dynamic calibration, time constant, quantum neural network, control algorithm

## Abstract

The aim of this study is to solve the problem of it being difficult to obtain quantitative step signals when testing the time constant of thermocouples using the laser excitation method, thereby restricting the accuracy and repeatability of the test of the time constant of thermocouples. This paper designs a thermocouple time constant testing system in which laser power can be adjusted in real time. The thermocouple to be tested and a colorimetric thermometer with a faster response speed are placed on a pair of conjugate focal points of an elliptic mirror. By taking advantage of the aberration-free imaging characteristic of the conjugate focus, the temperature measured by the colorimetric thermometer is taken as the true value on the surface of the thermocouple so as to adjust the output power of the laser in real time, make the output curve of the thermocouple reach a steady state, and calculate the time constant of the thermocouple. This paper simulates and analyzes the effects of adjusting PID parameters using quantum neural networks. By comparing this with the method of optimizing PID parameters with BP neural networks, the superiority of the designed QNN-PID controller is proven. The designed controller was applied to the test system, and the dynamic response curves of the thermocouple reaching equilibrium at the expected temperatures of 800 °C, 900 °C, 1000 °C, 1050 °C, and 1100 °C were obtained. Through calculation, it was obtained that the time constants of the tested thermocouples were all within 150 ms, proving that this system can be used for the time constant test of rapid thermocouples. This also provides a basis for the selection of thermocouples in other subsequent temperature tests. Meanwhile, repeated experiments were conducted on the thermocouple test system at 1000 °C, once again verifying the feasibility of the test system and the repeatability of the experiment.

## 1. Introduction

Temperature is a physical quantity that indicates the degree of hotness or coldness of an object. The accuracy of temperature measurement has extremely important practical significance in fields such as industrial production, agricultural production, and aerospace [1]. Temperature measurement, as an important fundamental means of modern technology, profoundly influences all fields of human production and life. Current temperature measurement methods can mainly be divided into two types: contact temperature measurement and non-contact temperature measurement [2,3,4,5]. Contact temperature measurement achieves temperature equilibrium through heat conduction between the temperature sensor and the measured component [6,7,8,9]. The advantages of this temperature measurement method lie in its relatively high accuracy and precision. The design structure of the contact temperature sensor is also relatively simple, with low cost, stable performance, and a wide temperature measurement range [10,11]. However, the drawback is that it requires full contact with the object under test, which leads to a change in the original temperature field of the object under test and requires the object under test to have a large heat capacity [12,13]. In non-contact temperature measurement, there is no need for direct contact between the temperature sensor and the object being measured. Instead, the temperature is measured by collecting physical quantities such as light waves emitted outward when the object undergoes heat exchange with the outside world. This does not change the temperature field of the object being measured, and the upper limit of temperature measurement is generally high [14,15]. However, since this temperature measurement method is easily affected by the surrounding environment during the temperature measurement process, and parameters such as the emissivity of the object need to be obtained at the same time, if the error of the obtained parameters is large and the temperature measurement distance is wrongly grasped, the accuracy of the same temperature field under different testing methods will decrease [16,17]. With the continuous research and development of large-range contact sensors, their limitations in the temperature measurement process have been greatly improved, and they are widely used in multiple fields such as aerospace, the military industry, and chemical engineering [18].

In contact temperature measurement, the thermocouple is one of the most suitable temperature sensors. It has the advantages of simple structural design, low cost, and convenient use [19]. The key to temperature measurement using thermocouples lies in achieving thermal equilibrium between the thermocouple and the object being measured. When dealing with objects with slow temperature changes, thermocouples can accurately measure temperature variations. However, when measuring objects with rapid temperature change frequencies, ordinary thermocouples cannot output the temperature in real time. At this point, it is necessary to select some thermocouples with faster response speeds [20]. The time constant of a thermocouple, as a core parameter characterizing the dynamic characteristics of the thermocouple [21], reflects the rate at which the sensor tracks temperature changes. It is an important reference for thermocouple selection. The time constants of different models of thermocouples are generally different, and even the time constants of the same model of thermocouple may vary in different temperature measurement environments [22]. Therefore, the time constant calculated by the equation method is inaccurate. If the accurate time constant of the thermocouple cannot be obtained, it will inevitably cause a series of problems for the measured temperature. Therefore, it is particularly important to accurately measure the time constant of the thermocouple through experiments, analyze its dynamic performance, and minimize its dynamic measurement error as much as possible.

Thermocouple time constant testing methods are usually classified according to the type of heat source. Common methods include the water bath method, flame method, hot wind tunnel method, laser method, shock tube method, etc. [23,24]. Among different types of thermal excitation sources, lasers have the advantages of fast application speed and adjustable parameters. Moreover, compared with other heat sources, laser temperature excitation can effectively reduce the errors in the process of applying thermal excitation. Therefore, the most commonly used scheme for thermocouple time constant testing experiments is to use a laser to excite the thermocouple, obtain the response curve, and calculate the time constant.

Xinming Su et al. established a dynamic response time test platform to test a NiCr/NiSi thin-film thermocouple [25]. An energy-adjustable pulsed laser was used as the excitation heat source. Meanwhile, a high-frequency infrared thermometer was employed to measure the instantaneous temperature variation curve of the coating surface of the thin-film thermocouple, and it was taken as the input signal for dynamic calibration. Li Yanfeng et al. established a dynamic test system for thermocouples [26], using a high-power laser to generate pulse excitation. They conducted dynamic response tests on K-type thermocouples using this system and analyzed the dynamic characteristics of the thermocouples by adopting the system identification method.

Although the thermocouple achieved rapid heating through the above-mentioned test method, due to the constant laser power, the temperature value of the thermocouple kept rising and did not stabilize around a certain value, resulting in the inaccurate calculation of the thermocouple time constant. Therefore, in this article, a thermocouple time constant testing system for dynamically adjusting the output power of the laser is designed. Utilizing the optical characteristics of the ellipsoidal mirror, a colorimetric thermometer with a response speed much faster than that of the thermocouple is adopted to dynamically calibrate the measured temperature values of the thermocouple. Meanwhile, a QNN-PID controller was introduced in the power control of the laser, and the PID parameters were adjusted by using the quantum neural network, which improved the control accuracy of the laser power. Through this system, the thermocouple can achieve a relatively ideal step temperature rise. On this basis, the time constant of the thermocouple can be calculated to judge its dynamic performance.

## 2. Materials and Methods

### 2.1. Theory of Thermocouple Time Constant Testing

When the measuring end of the thermocouple is in thermal equilibrium, the thermal equilibrium equations of the measuring end are as shown in Equations (1)–(5).

(1) $$q_{s}=q_{c}+q_{r}+q_{h}$$

(2) $$q_{s}=\rho C_{P}V\frac{dT_{j}}{\mathrm{dt}}$$

(3) $$q_{c}=\mathrm{aA}\left(T_{g}-T_{j}\right)$$

(4) $$q_{r}=\varepsilon C_{0}A\left[{\left(\frac{T_{j}}{100}\right)}^{4}-{\left(\frac{T_{w}}{100}\right)}^{4}\right]$$

(5) $$q_{h}=\frac{\pi D^{2}}{4}k_{m}\frac{T_{b}-T_{j}}{l}$$

In these equations, q_s_ is the heat storage at the measuring end, q_c_ is the heat transfer at the measuring end, q_r_ is the radiative heat transfer at the measuring end, q_h_ is the heat transfer conducted from the thermocouple wire to the measuring end, ρ is the density at the measuring end, C_p_ is the specific heat at the measuring end, V is the volume at the measuring end, a is the convective heat transfer coefficient, A is the effective contact area between the measuring end of the thermocouple and the fluid, T_j_ is the temperature at the measuring end, T_g_ is the medium temperature, and ε is the emissivity at the measuring end. C_0_ is the absolute blackbody radiation coefficient, k_m_ is the thermal conductivity of the couple wire, T_b_ is the temperature of the couple wire at a distance l from the measurement end, and l is the length of the couple wire.

In a general sense, a thermocouple whose transfer function is treated as first-order refers to a thermocouple that ignores two terms on the right side of Equation (1), namely the heat conduction and radiation at the temperature measurement end of the thermocouple, as shown in Equation (6). By substituting Equations (2) and (3) into Equation (6) and converting them, Equations (7) and (8) can be obtained.

(6) $$q_{s}=q_{c}$$

(7) $$\rho C_{P}V\frac{dT_{j}}{\mathrm{dt}}=\mathrm{aA}\left(T_{g}-T_{j}\right)$$

(8) $$\frac{\rho C_{P}V}{\mathrm{aA}}\frac{dT_{j}}{\mathrm{dt}}+T_{j}=T_{g}$$

Let $\tau =\frac{\rho C_{P}V}{\mathrm{aA}}$. According to Equation (8), Equation (9) can be obtained.

(9) $$\tau \frac{dT_{j}}{\mathrm{dt}}+T_{j}=T_{g}$$

Equation (9) is a first-order linear differential equation, and its general solution is shown in Equation (10).

(10) $$T_{j}=Ce^{-\frac{t}{\tau }}+T_{g}$$

In this equation, C is the integral constant, which is determined by the initial conditions. Suppose at t = 0, $T_{j}=T_{j0}$, and then Equations (11)–(13) can be obtained.

(11) $$T_{j0}=Ce^{-\frac{t}{\tau }}+T_{g}=C+T_{g}$$

(12) $$T_{j}=\left(T_{j0}-T_{g}\right)e^{-\frac{t}{\tau }}+T_{g}$$

(13) $$\frac{T_{j}-T_{j0}}{T_{g}-T_{j0}}=1-e^{-\frac{t}{\tau }}$$

According to Equation (13), when t equals τ, the indicated temperature of the thermocouple reaches 63.2% of the step temperature, which is the definition of the thermocouple time constant.

**Figure 1:**
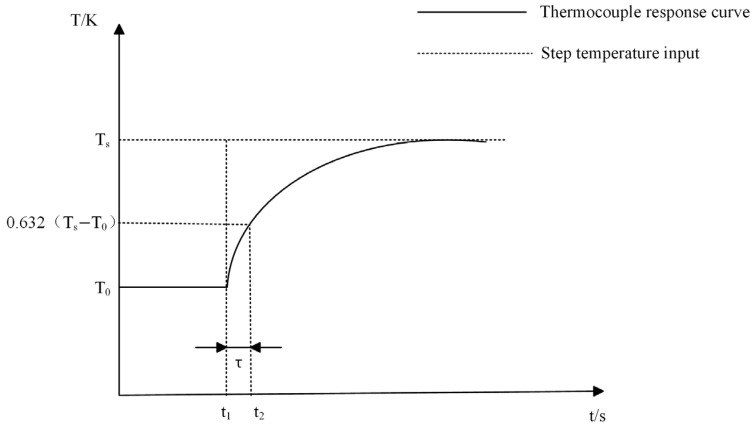
Step response curve of thermocouple.

Basic methods for testing the time constant of thermocouples involve forming a quantitative temperature step change at the temperature measurement end of the thermocouple and then collecting and plotting the output data graph at the output end of the thermocouple. The ideal temperature–time curve of the thermocouple after being excited by the step temperature is shown in Figure 1, where T_s_ is the stable step temperature, and T_0_ is the initial temperature.

Furthermore, the selection of the response termination point of the measured thermocouple to the step temperature determines the total step temperature quantity and directly affects the τ value. Many studies have shown that when  t≥5τ, it can be regarded as $T_{j}=T_{g}$; that is, the step temperature tends to equilibrium after 5τ. Therefore, the stable duration for the thermocouple to reach the expected step temperature needs to be greater than 5τ in order to obtain an accurate time constant value.

If the heat conduction and radiative heat transfer at the temperature measurement end of the thermocouple are not ignored, Equation (1) is an extremely complex thermal equilibrium equation, and it is very difficult to obtain its general solution. In addition, for armored thermocouples with unexposed nodes and thermocouples with protective sleeves, when considering the thermal equilibrium of the thermocouple, it is also necessary to take into account that the equations for the thermal equilibrium caused by different shell materials are not the same, and the process is more complex. This makes it difficult to theoretically calculate the time constant of the thermocouple. From the above content, it can be concluded that the time constant value of a thermocouple, that is, the dynamic response speed of the thermocouple, is closely related to its own material properties, three-dimensional structure, and convective heat transfer coefficient. However, these characteristic parameters of the thermocouple are not constant. Under different experimental environments, these values may change with air pressure, temperature, humidity, and the inherent properties of the object being measured. Therefore, the acquisition of the time constant of a thermocouple is specific to the environment. The time constant of a thermocouple measured in a low-temperature and low-speed environment is highly likely not applicable to a transient high-temperature environment. To address this issue, it is particularly necessary to establish a comprehensive thermocouple time constant testing system to ensure that the selection of thermocouples can be completed in different testing environments.

### 2.2. Thermocouple Time Constant Testing System

In the laser excitation method, when a laser is used to heat a thermocouple, if the power output of the laser is fixed, since the heating power of the laser is greater than the heat dissipation power of the thermocouple, the energy output by the laser accumulates on the surface of the thermocouple. As the heating time increases, the temperature on the surface of the sensor will also continuously rise, as shown in Table 1, where P_h_ represents the heating power of the thermocouple being measured. P_d_ represents the heat dissipation power of the thermocouple under test. In order to achieve the precise control of the surface temperature of the thermocouple, it is essentially necessary to establish a dynamic thermal balance mechanism. When the thermocouple’s temperature reaches the preset target value, the test system initiates a power regulation protocol: the laser control module dynamically adjusts the laser output power to maintain equilibrium between the Joule heating power generated through laser excitation and the heat dissipation power from the material surface via conduction, convection, and radiation. In this way, the thermocouple will remain in a thermal equilibrium state and maintain a fixed temperature. Therefore, this thesis proposes to adjust the laser power by using the negative feedback method to form an instantaneously balanced temperature on the surface of the thermocouple.

**Figure 2:**
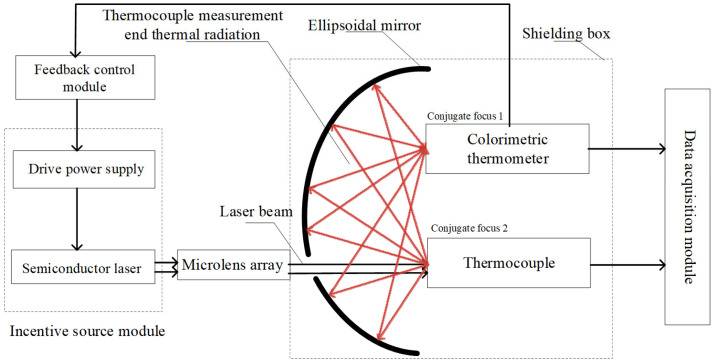
Schematic diagram of thermocouple time constant test system.

**Figure 3:**
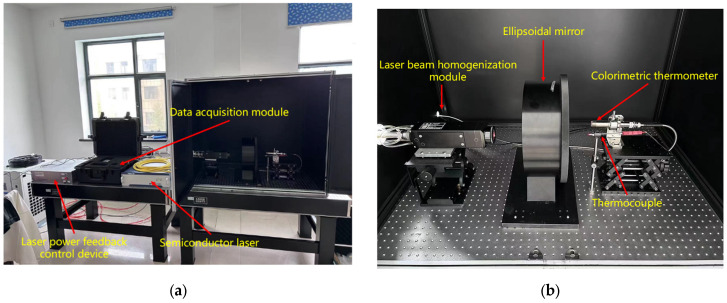
Physical diagram of thermocouple constant test system; (**a**) overview diagram of thermocouple time constant testing system; (**b**) diagram of internal device of shielding box.

The thermocouple time constant testing system used in this thesis mainly consists of a semiconductor laser module (Wuhan Ruike Laser Technology Co., LTD., Wuhan, China), a laser power feedback control module (Self-made), a data acquisition module (Shanghai Jianyi Technology Co., LTD., Shanghai, China), an ellipsoidal mirror module (Self-made), a colorimetric temperature measurement module (Changzhou Sijie Optoelectronic Technology Co., LTD., Changzhou, China), etc. A schematic diagram of the testing system is shown in Figure 2, and a physical diagram of the testing system is shown in Figure 3.

During the experiment, the semiconductor laser was used as the step excitation source of the thermocouple under test. Through the Settings of its driving software, the output power of the laser could be changed, and the temperature step at the temperature measurement end of the thermocouple could be achieved in a very short time. To prevent harm from laser reflections and eliminate interference from ambient light on the measurement of the colorimetric temperature measurement module, the colorimetric temperature measurement module, the measured thermocouple, and the ellipsoidal mirror need to be placed in a shielded box. According to the indicator red light of the laser, the laser emitted by the laser is placed in a straight line with the opening of the shield box and the thermocouple to be measured. The thermocouple to be measured and the colorimetric temperature measurement module are placed at the two conjugate foci of the ellipsoidal mirror. After the thermocouple is excited by the laser, the ellipsoidal mirror can converge the radiation energy it generates to the colorimetric temperature measurement module. Because the colorimetric temperature measurement module responds much faster than the thermocouple, its reading can be considered the true surface temperature of the thermocouple. This value is then input to the control module via an Analog-to-digital converter. The control module subsequently adjusts the laser’s output power through feedback control to achieve the set temperature on the thermocouple’s surface. The data acquisition module collects the time–voltage (t−V) response curve of the thermocouple during operation, calculates the collected voltage value according to the corresponding scale table of the thermocouple used, converts it into the temperature value of the thermocouple, and finally obtains the time constant of the thermocouple under different control strategies by analyzing the time–temperature (t−T) curve of the thermocouple.

**Figure 4:**
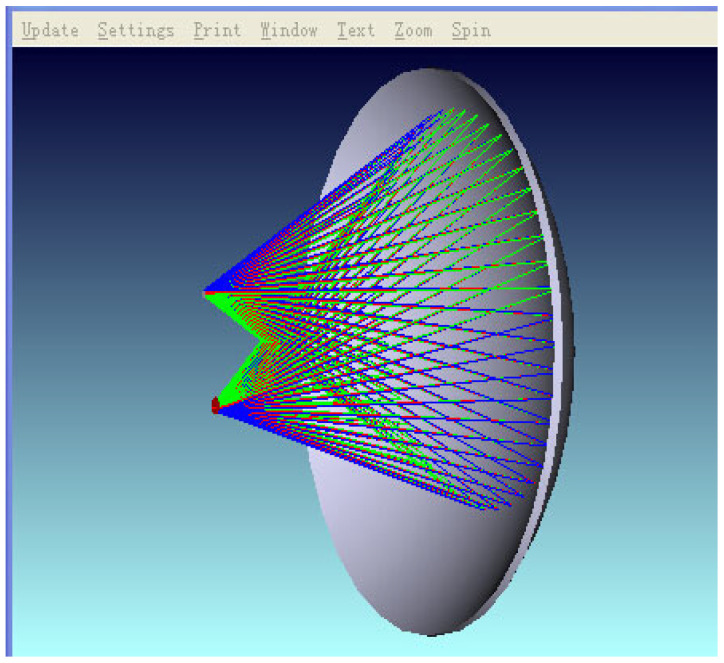
A simulation of the distance between the two conjugate focal points of the elliptic mirror.

In this testing system, the most important component is the ellipsoidal mirror module. When heating a thermocouple using the laser method, the thermocouple junction exhibits a unique heat dissipation behavior during the laser energy injection process: its microscale structure significantly limits the heat capacity. When the surface temperature rise in the thermocouple is in a relatively low range, the heat exchange between the junction and the surrounding environment presents a quasi-point source radiation mode, and the thermal energy is uniformly dispersed into three-dimensional space in an approximately spherical wave form. If a colorimetric thermometer is used to directly measure the surface temperature of the thermocouple under test, it is prone to interference from other surrounding heat sources, resulting in inaccurate output. Therefore, it is necessary to converge the thermal radiation on the surface of the thermocouple under test. The ellipsoidal focusing mirror is an inwardly concave mirror, which has relatively ideal conjugate imaging properties and can provide valuable optical characteristics: that is, when an object is at any one of the focal points, after reflection, it can be imaged without aberration at the other focal point. In order to give full play to its conjugate imaging characteristics, it is obtained through theoretical calculation that when the radius of the major axis is 177 mm, the radius of the major axis is 170 mm, and the distance between the conjugate foci is 49.28 mm, the sphericity of the ellipsoidal mirror is not less than 3sr, and the PV value of the ellipsoidal focusing mirror is 8 μm. This is simulated by using ZEMAX software2009. A simulation shadow diagram is shown in Figure 4. It is found that at this time, there is a better reflection and convergence effect, which can meet the test requirements of the system designed in this thesis.

**Figure 5:**
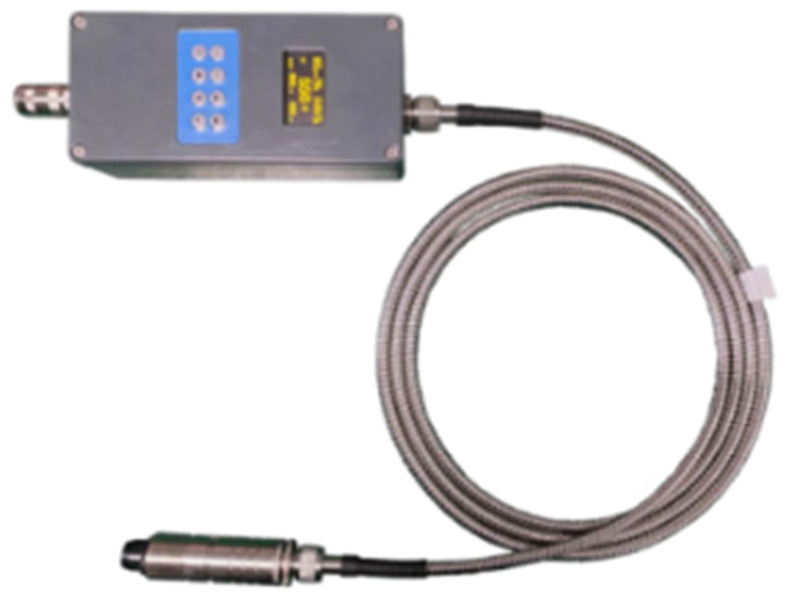
A physical picture of the colorimetric thermometer.

When choosing the appropriate temperature measurement module, the first thing to consider is that the response speed of the temperature measurement module must be faster than that of the thermocouple so that it can be used as the calibration source of the thermocouple. Secondly, the output of the temperature measurement module should have at least two channels, simultaneously fulfilling the functions of data acquisition and serving as the input signal for the feedback controller. Finally, due to the testing requirements, the temperature measurement module needs to have the characteristic of high-temperature resistance. Therefore, the temperature measurement module used in the system designed in this paper is a high-performance and intelligent colorimetric optical fiber infrared thermometer. It is composed of a lens, optical fiber, and processing components. The optical fiber and lens assembly can withstand a high temperature of 250 °C without additional cooling. It adopts a stainless steel lens, an aluminum diecast housing, and a protection grade of IP65. A physical diagram of the colorimetric thermometer is shown in Figure 5, and its main parameters are presented in Table 2.

In the system designed in this paper, the colorimetric thermometer is the calibration source of the thermocouple to be measured. Therefore, the upper limit of the thermocouple time constant that this system can test is consistent with the response speed of the colorimetric thermometer, which is 1 ms.

### 2.3. PID Controller Based on Quantum Neural Network

In the control system designed in this thesis, the application of the feedback control algorithm can lead to the better formation of a step temperature rise on the surface of the thermocouple. PID control is the most fundamental method in feedback control algorithms. It takes the deviation between the system set value and the output value as the system input and outputs the control quantity through proportional, integral, and differential calculations to complete the control of the controlled object. The PID control algorithm is widely used in engineering practice due to its advantages such as mature technology, good reliability, and simple structure. However, for nonlinear time-varying uncertain systems, the control effect of the PID control algorithm is not ideal. Moreover, in practical engineering applications, the adjustment of PID parameters is mostly based on experience for trial matching, which undoubtedly increases the difficulty and workload of the experiment. Therefore, in this article, quantum neural networks are used to conduct the real-time dynamic adjustment of PID control parameters, with the expectation of achieving good control effects for nonlinear systems.

**Figure 6:**
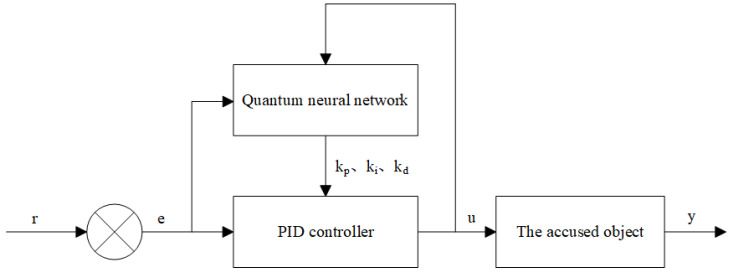
PID controller structure based on quantum neural network.

The structure of the PID control system based on the quantum neural network is shown in Figure 6. The controller consists of two parts: One is the conventional PID controller, which is used to directly perform closed-loop control on the object, and the three parameters are tuned online. The second is the quantum neural network. According to the operating state of the system, through the information calculation method of the neurons in the quantum neural network and the adjustment of the weight coefficient matrix based on the gradient descent method, the output variables of the quantum neural network correspond to the parameters of the PID controller under the optimal control rate, and thus the PID parameters are adjusted to achieve the optimization of certain performance indicators.

The algorithm process of the PID controller based on the quantum neural network can be summarized as follows:

➀ The structure of the quantum neural network is determined; that is, the number of nodes in the input layer and the hidden layer is determined, and the initial values of each variable are determined, setting k = 1.

➁ The system obtains r(k) and y(k) through sampling and calculates the error e(k) at this moment = r(k) − y(k).

➂ The input and output of neurons in each layer are calculated. In this calculation, the output of the output layer of the quantum neural network consists of the three adjustable parameters k_p_, k_i_, and k_d_ of the PID controller.

➃ The output u(k) of the PID controller based on the PID control algorithm is calculated.

➄ The learning of quantum neural networks is conducted, the parameters of the quantum neural networks are adjusted online, and the adaptive adjustment of PID control parameters is achieved.

➅ Let k = k + 1, and ➀ is returned.

Since the system output variables k_p_, k_i_, and k_d_ are all non-negative real values, the selection of the output layer activation function must strictly follow the physical feasible domain criterion of the control parameters. In order to have both constraint completeness and convergence smoothness, the activation function from the hidden layer neurons to the output layer neurons adopts a non-negative Sigmoid function, and its mathematical expression is shown in Equation (14).

(14) $$f(x)=\frac{1}{2}(1+tanh(x))=\frac{e^{x}}{e^{x}+e^{-x}}$$

**Figure 7:**
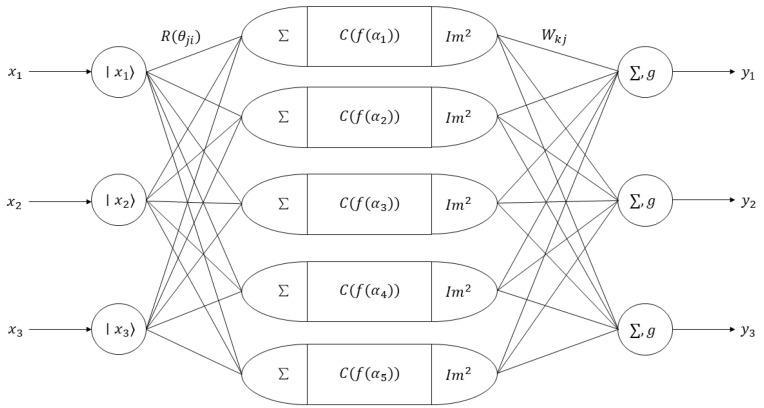
Schematic diagram of quantum neural network structure.

The quantum neural network designed in this thesis is composed of an input layer, a hidden layer, and an output layer. Among these layers, the hidden layer is set as a single-layer network. This is because in application practice, when the number of hidden layers is set to one or two, the convergence characteristics of the network will be better. Poor convergence occurs when the number of layers in the network’s hidden layer is two or more or when there are none. Since the designed quantum neural network is used to tune the PID control parameters, the input variables of the network are the error of the control system, the integral of the error, and the differential of the error. Therefore, the number of neurons in the input layer is 3. The output of the network consists of the coefficients of the proportion, integral, and differential. Therefore, the number of neurons in the output layer is also 3. The number of neurons in the hidden layer is selected by the trial-and-error method. When the number of neurons in the hidden layer is 5, the systematic error is the smallest. Therefore, the number of neurons in the hidden layer is determined to be 5. The structure of the designed quantum neural network is shown in Figure 7.

In practical applications, the input variables of neural networks are often real values and need to be transformed into quantum state inputs $|x_{i}\rangle$. The variable transformation formula is as shown in Equation (15).

(15) $$|x_{i}\rangle =\cos(\frac{2\pi }{1+e^{-x_{i}}})|\;0\rangle +\sin(\frac{2\pi }{1+e^{-x_{i}}})|\;1\rangle ={\left[\begin{array}{cc} \cos(\frac{2\pi }{1+e^{-x_{i}}}) & \sin(\frac{2\pi }{1+e^{-x_{i}}}) \end{array}\right]}^{\prime }$$

Quantum rotation gates, represented by R(θ), are one of the main ways to transform quantum states and are defined as in Equation (16).

(16) $$R(\theta )=\left[\begin{array}{cc} \cos(\theta ) & -\sin(\theta )\\ \sin(\theta ) & \cos(\theta ) \end{array}\right]$$

For the input quantum state |x〉, R(θ) acting on |x〉 can yield Equation (17).

(17) $$R(\theta )|x_{i}\rangle =\left[\begin{array}{cc} \cos(\theta ) & -\sin(\theta )\\ \sin(\theta ) & \cos(\theta ) \end{array}\right]\left[\begin{array}{c} \cos(\alpha )\\ \sin(\alpha ) \end{array}\right]=\left[\begin{array}{c} \cos(\theta +\alpha )\\ \sin(\theta +\alpha ) \end{array}\right]$$

After the quantum states of the input variables are rotated, the aggregation operation can be expressed as Equation (18).

(18) $$\sum_{i=1}^{n}R(\theta_{i})|x_{i}\rangle ={\left[\begin{array}{cc} \cos(\theta ) & \sin(\theta ) \end{array}\right]}^{\prime }$$

After this aggregation operation, the output result of the hidden layer can complete the flipping operation after the controlled not gate action. The representation of the controlled not gate is shown in Equation (19).

(19) $$C(t)=\left[\begin{array}{cc} \cos(\frac{\pi }{2}t-2\alpha ) & -\sin(\frac{\pi }{2}t-2\alpha )\\ \sin(\frac{\pi }{2}t-2\alpha ) & \cos(\frac{\pi }{2}t-2\alpha ) \end{array}\right]$$

For the input quantum state $|x\rangle ={\left[\begin{array}{cc} \cos(\alpha ) & \sin(\alpha ) \end{array}\right]}^{\prime }$, the action of C(t) on |x〉 yields Equation (20).

(20) $$C(t)|x\rangle =\left[\begin{array}{cc} \cos(\frac{\pi }{2}t-2\alpha ) & -\sin(\frac{\pi }{2}t-2\alpha )\\ \sin(\frac{\pi }{2}t-2\alpha ) & \cos(\frac{\pi }{2}t-2\alpha ) \end{array}\right]\left[\begin{array}{c} \cos(\alpha )\\ \sin(\alpha ) \end{array}\right]=\left[\begin{array}{c} \cos(\frac{\pi }{2}t-\alpha )\\ \sin(\frac{\pi }{2}t-\alpha ) \end{array}\right]$$

When t = 1, $C(t)|x\rangle ={\left[\begin{array}{cc} \sin(\alpha ) & \cos(\alpha ) \end{array}\right]}^{\prime }$, causing the phase of |x〉 to flip, and the quantum state output of the hidden layer neuron is shown in Equation (21).

(21) $$|h_{j}\rangle =\left[\begin{array}{c} \cos\left(\frac{\pi }{2}f\left(\alpha_{j}\right)-\arctan\left(\sum_{i=1}^{n}R(\theta_{\mathrm{ji}})|x_{i}\rangle \right)\right)\\ -\sin\left(\frac{\pi }{2}f\left(\alpha_{j}\right)-\arctan\left(\sum_{i=1}^{n}R(\theta_{\mathrm{ji}})|x_{i}\rangle \right)\right) \end{array}\right]$$

Here, f (·) is the activation function, and the actual output of the hidden layer neurons is the probability amplitude of the quantum ground state |1〉. Therefore, the output of the hidden layer is shown in Equation (22).

(22) $$h_{j}=\sin^{2}\left(\frac{\pi }{2}f\left(\alpha_{j}\right)-\arctan\left(\sum_{i=1}^{n}R(\theta_{\mathrm{ji}})|x_{i}\rangle \right)\right)$$

The output of the output layer is shown as Equation (23).

(23) $$y_{k}=g\left(\sum_{j=1}^{p}w_{kj}h_{j}-b_{k}\right)=g\left(\sum_{j=1}^{p}w_{kj}\mathrm{si}n^{2}\left(\frac{\pi }{2}f\left(\alpha_{j}\right)-\arctan\left(\sum_{i=1}^{n}R(\theta_{i})|x_{i}\rangle \right)\right)-b_{k}\right)$$

In this formula, g (·) is the activation function of the output layer; i = 1, 2, …, n; j = 1, 2, …, p; and k = 1, 2, …, m, where n is the number of neurons in the input layer, which is equal to 3; p is the number of neurons in the hidden layer, which is equal to 5; and m is the number of neurons in the output layer, which is equal to 3.

In the general training process of PID parameters in neural networks, the loss function is selected as the integral square error of the system response, with the goal of minimizing the integral of the square of the error over the entire time domain. However, in this thermocouple time constant test system, in order to prevent excessive control quantity parameters and excessive system output overshoot, the loss function needs to be improved. The original loss function and the improved loss function are shown in Equations (24)–(27).

(24) $$L_{\mathrm{total}}=L_{1}+L_{2}+L_{3}$$

(25) $$L_{1}=\int_{t_{0}}^{t_{f}}e(t{)}^{2}\mathrm{dt}$$

(26) $$L_{2}=\lambda_{1}{\int_{t_{0}}^{t_{f}}\left(\frac{\mathrm{de}(t)}{\mathrm{dt}}\right)}^{2}$$

(27) $$L_{3}=\lambda_{2}\int_{t_{0}}^{t_{f}}\max\left(0,\;y\left(t\right)-r\right)\mathrm{dt}$$

In these equations, $e\left(t\right)=r-y\left(t\right)$, which is used to track the error between the system output and the set value. $\lambda_{1}=0.1$ represents the penalty coefficient, and $\lambda_{2}=10$ represents the overshoot penalty weight.

The original loss function is L_1_, which can minimize the steady-state error, enable the system to quickly convergence to the set value, and shorten the regulation time of the system. The improved loss function adds the error differential penalty term L_2_ and the overcall penalty term L_3_. The error differential penalty term can suppress the rapid change in the error, reduce the high-frequency oscillation of the system, and significantly lower the time constant of the control system. The overshoot penalty term imposes a penalty when the system output exceeds the set value, suppressing the system output overshoot and enhancing the stability of the system.

## 3. Results

### 3.1. Analysis of QNN-PID Controller Simulation Results

**Figure 8:**
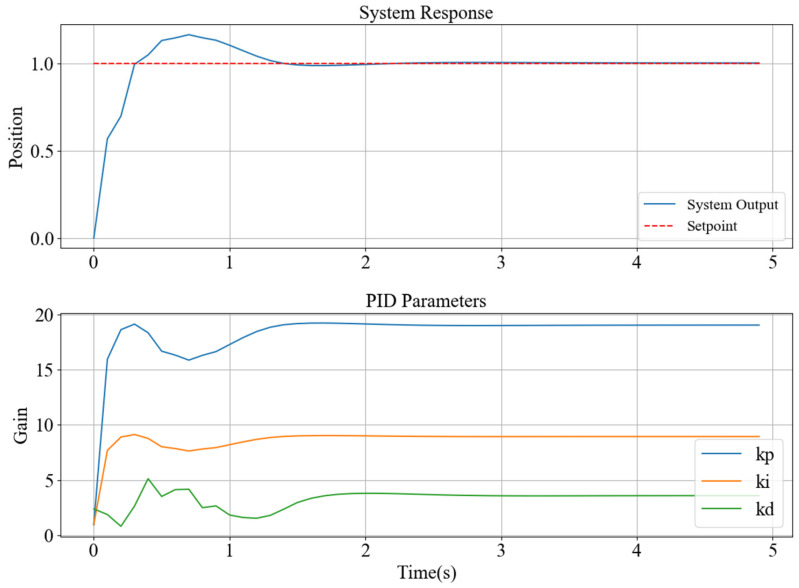
QNN-PID control simulation output curve and PID parameter change curve.

Taking the second-order system $G\left(s\right)=\frac{0.227s+0.8779}{s^{2}+0.499s+0.5788}$ as an example of the simulation system, the output response setting value of the constructed quantum neural network PID controller is 1, the number of neurons in the input layer is 3, the number of neurons in the hidden layer is 5, and the number of neurons in the output layer is 3. The maximum number of training times is 1000, the simulation time is 5 s, the variation range of k_p_ is [0, 20], the variation range of k_i_ is [0, 10], and the variation range of k_d_ is [0, 6]. Under these conditions, the response curve, PID parameter variation curve, and loss function variation curve of the simulation system were obtained, as shown in Figure 8.

It can be seen from the response curve of the simulation system that the maximum output amplitude of the simulation system is 1.151, the time to reach the steady state is 1.430 s, and the time to reach the peak is 0.556 s. The time constant of the simulation system under QNN-PID control is calculated to be 351 ms. Meanwhile, the steady-state error of the simulation system approaches 0. The output of the stable system is no different from the set value, which indicates that the quantum neural network can be used to quickly adjust the parameters of the PID controller, thereby enabling the system to quickly reach the steady-state value, and the overshoot of the simulation system can be controlled within 15%.

It can be seen from the PID parameter variation curve that in the early stage of network training, k_p_, k_i_, and k_d_ are significantly adjusted within the given range to find the parameters that make the system output reach the set value. After 1 s, the variation trend in PID parameters decreases, and the output curve of the system also tends to be stable. Finally, the parameters of the PID controller are k_p_ = 19.06, k_i_ = 8.85, and k_d_ = 3.65.

**Figure 9:**
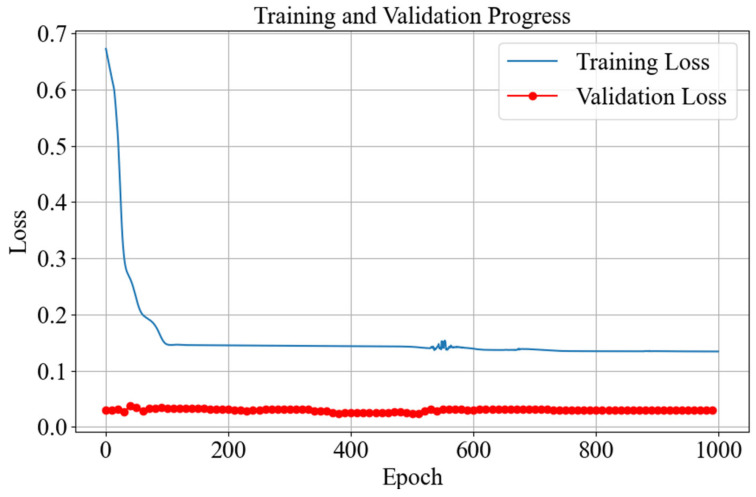
The variation curves of the loss function between the training set and the validation set.

In this simulation experiment, the input of the training set is the error signal of the system under the zero initial condition (including systematic error, integral of error, and differential of error), and the input of the verification set is the systematic error signal under the non-zero initial condition. It can be seen from Figure 9 that near the 100th training, the loss function of the network reaches the minimum value, indicating that the convergence speed of the network is very fast and the PID parameter values that stabilize the system can be found quickly. The final value of the loss function of the training set in the figure is 0.1341, meeting the convergence requirements. It can be seen from the variation curve of the loss function of the validation set that there are some fluctuations in the loss function at the beginning of training, but it also met the convergence requirements. Finally, the value of the loss function of the validation set is 0.0298, which indicates that this network has good robustness and still has a good generalization ability for the unseen initial conditions and can eliminate the state disturbances of the controller well.

**Figure 10:**
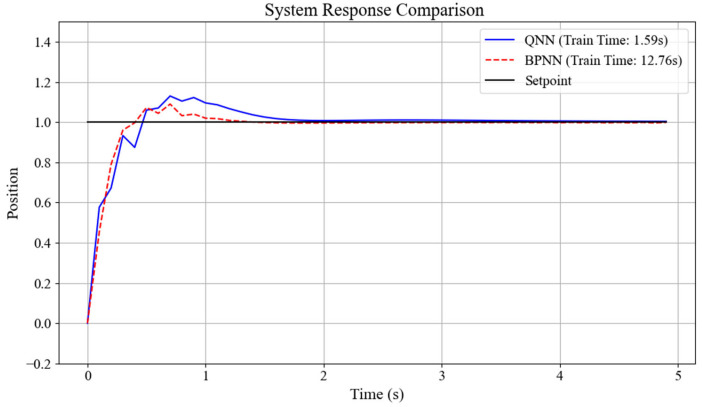
Comparison chart of output responses between QNN and BPNN.

**Figure 11:**
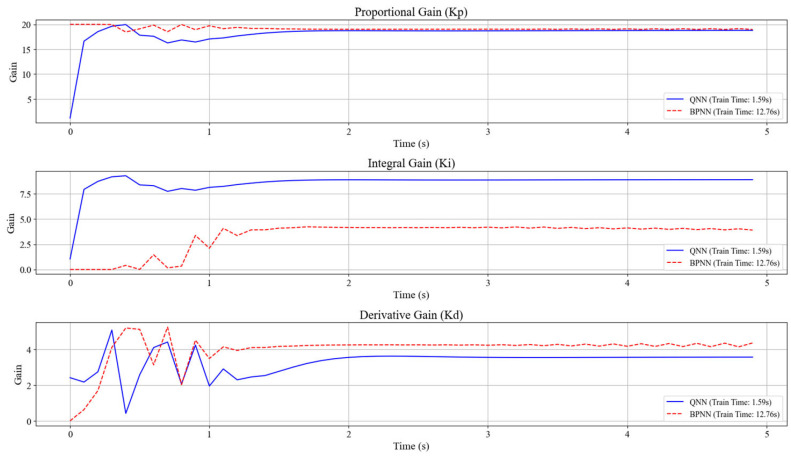
Comparison chart of PID parameter variation curves between QNN and BPNN.

To verify the superiority of the designed network, the results of the quantum neural network and the BP neural network were compared. The BP neural network used in contrast has three neurons in the input layer, five neurons in the hidden layer, and three neurons in the output layer, which is consistent with the quantum neural network. Meanwhile, the input and output of the network are also consistent with those of the quantum neural network. The comparison graphs of the output responses and PID parameter variation curves of the two networks are obtained, as shown in Figure 10 and Figure 11.

It can be seen from the output curves of the simulation system in Figure 10 that under the control of two different network PID controllers, the time for the simulation system to reach the peak is basically the same, both being about 0.7 s, indicating that both controllers have a fast response speed and excellent dynamic performance. Moreover, the systems eventually reached the steady state, and the steady-state errors were all within 2%, indicating that both controllers have excellent stability and robustness, and the tracking accuracy is very high. However, the training durations of the two networks are significantly different. The training time of the quantum neural network is 1.59 s, while that of the BP neural network is 12.76 s. This indicates that the designed quantum neural network is significantly more efficient than the BP neural network and has certain superiority in computational efficiency.

It can be concluded from the PID parameter variation curve in Figure 11 that both networks can quickly obtain stable final values of PID parameters. Although the obtained final values of parameters are different, this is reasonable. Because for a dynamic system, the combination of PID parameters that enables it to achieve good performance is often more than one, the neural network only obtains the optimal combination of PID parameters at this time when the loss function is minimized.

### 3.2. Test Experiments of Thermocouple Time Constants at Different Temperatures

**Figure 12:**
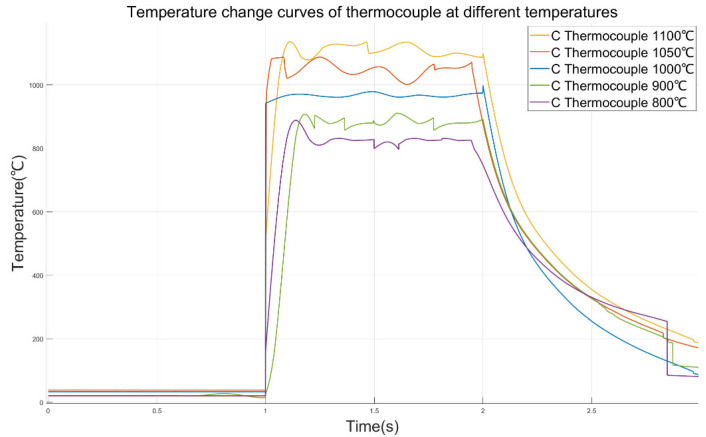
Temperature change curves of thermocouples at different temperatures.

The C-type thermocouple used in the test in this paper is the E6-20 Series C-type thermocouple produced by Nanmac Company (397 Williams St, Marlborough, MA, The United States of America). Its diameter is 0.508 mm, and it has a three-layer insulation structure. When applying the system constructed in this thesis to test the time constant of thermocouples, a laser is output at 1 s. The sampling frequency of the thermocouple is set to 2000 Hz. The temperature values measured by the colorimetric thermometer and the thermocouple are taken as the inputs of the quantum neural network to obtain the optimal parameters of the PID controller. The obtained kp, ki, and kd are applied to the PID feedback control circuit. The laser output power was changed in real time to make the C-type thermocouple reach temperature equilibrium at 800 °C, 900 °C, 1000 °C, 1050 °C, and 1100 °C, and the temperature variation curve of the thermocouple was obtained, as shown in Figure 12.

It can be seen from the curve that in the experiment of raising the temperature of the thermocouple to 1000 °C, the temperature of the thermocouple suddenly rose. This might be because during the signal transmission process, the laser starts to output from 100% power and quickly reaches the set temperature value. Moreover, the sampling rate of the thermocouple data is relatively high, and a complete signal is recorded. Therefore, it is reasonable for the temperature of the thermocouple to rise rapidly within 1 ms. In the following period of time, the output curve of the thermocouple reached a stable state. This also indicates that the applied QNN-PID controller can easily enable the thermocouple to obtain an approximately ideal step temperature rise, and the measured time constant of the thermocouple is the closest to the true value. It can be obtained from the data in Table 3 that the maximum overprint of the thermocouple temperature curve under the control of the QNN-PID controller is 11.03%. This indicates that under the control of this controller, there will be no situation where the initial power of the laser is too large, causing damage to the thermocouple nodules, ensuring that the same thermocouple can be tested repeatedly multiple times in this system. The peak times were all achieved within 200 ms of the light output. This indicates that when the initial power of the laser is very small, the designed QNN-PID controller can rapidly increase the power of the laser, thereby enabling the temperature of the thermocouple to quickly reach the peak and gradually stabilize near the set value, which meets the requirement of a fast signal when designing the time constant test of the thermocouple. It should be noted that there is a small steep peak that suddenly rises at 2 s on the 1000 °C curve in the figure, but its value does not exceed 1000 °C. Therefore, it can be judged that there is no overshoot on the 1000 °C curve.

### 3.3. Repeatability Test of Thermocouple Time Constant at 1000 °C

**Figure 13:**
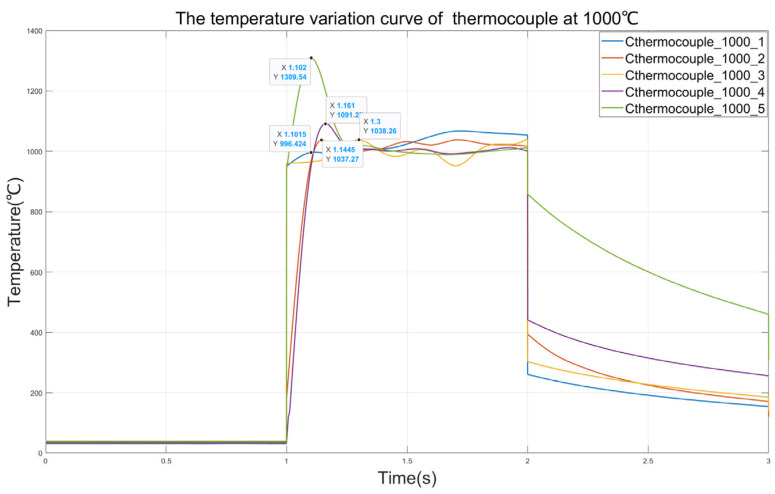
Temperature variation curve of thermocouple at 1000 °C.

To explore the repeatability of the thermocouple time constant testing system, five data points of the C-type thermocouple reaching temperature equilibrium at 1000 °C are obtained through experiments. Similarly to the previous experiments, the laser begins to produce output at 1 s, and the sampling rate of the thermocouple is 2000 Hz. The thermocouple temperature variation curve as shown in Figure 13 is obtained.

It can be seen from Figure 13 that in the five measurements at 1000 °C, the output curves of the thermocouple can all remain stable, which indicates the superiority of the QNN-PID controller. In the first test, the peak temperature of the thermocouple reached 1309 °C. This might be because in this test, the k_p_ value of the initial output of the QNN-PID controller was too large, resulting in a large overshoot of the thermocouple. In the third test, it can be observed that the response of the thermocouple is significantly slower than in the other several tests. This might be because in this test, the k_p_ value of the initial output of the QNN-PID controller is too small, resulting in a slow response of the thermocouple and a long rise time. It can be seen from Table 4 that, except for the data of the third thermocouple responding too slowly at 1000 °C, the time constants of the thermocouples obtained in the other tests are all around 100 ms, which is consistent with the time constants obtained in the previously mentioned experiments. This indicates that the repeatability of the system in testing the time constants of thermocouples is reliable.

## 4. Conclusions

In conclusion, this paper designs a thermocouple time constant test system for the feedback control of the output power of a laser and optimizes the parameters of the PID controller by using a quantum neural network. Combining quantum neural networks with PID control, a QNN-PID controller was designed. The response curve of the second-order system under the control of the QNN-PID controller was obtained through simulation, verifying the feasibility of this method. And by comparing it with the BPNN-PID controller, the superiority of the QNN-PID controller was proven. The QNN-PID controller was applied to the constructed thermocouple time constant test system. The experiment obtained the stable output curves of the C-type thermocouple under five working conditions of 800 °C, 900 °C, 1000 °C, 1050 °C, and 1100 °C. Through calculation, it was obtained that the time constant of the thermocouple under each working condition is within 150 ms, verifying that the designed system can meet the testing requirements of rapid thermocouples. Moreover, five repeated tests were conducted at 1000 °C, and the measured times of the C-type thermocouple were all within 150 ms, which proved the repeatability of this system for the time constant test of thermocouples. The preliminary tests of thermocouples using this system can provide support for the selection of thermocouples with appropriate dynamic performance in different subsequent test environments.

## Figures and Tables

**Table 1:** Influence of feedback control on output curve of thermocouple.

Laser Heating Power	The Temperature Variation in Thermocouples Under Non-Feedback Control	The Temperature Variation in Thermocouples Under Feedback Control
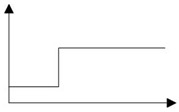	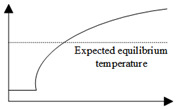 P_h_ > P_d_	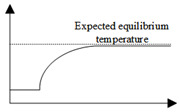 P_h_ = P_d_

**Table 2:** Main technical indicators of colorimetric temperature measuring instrument.

Parameter Type	Technical Indicators
Temperature measurement range	450~2500 °C
Response time	1 ms
Output resolution	16 bit
Control output	0~10 V
Number of output channels	3
Focusing mode	Manual
Focal length range	0.15 m to infinity
Communication interface	RS485, RS232

**Table 3:** Parameter values of temperature change curves of thermocouple at different temperatures.

Preset Temperature	Peak Temperature	Overshoot	Peak Time	Time Constant
800 °C	888.28 °C	11.03%	1.134 s	84.69 ms
900 °C	907.80 °C	0.87%	1.177 s	111.86 ms
1000 °C	970.64 °C	none	1.130 s	82.16 ms
1050 °C	1083.49 °C	3.19%	1.028 s	17.70 ms
1100 °C	1136.28 °C	3.29%	1.115 s	72.68 ms

**Table 4:** The parameter values of the thermocouple temperature change curve at 1000 °C.

Number of Experiments	Peak Temperature	Overshoot	Peak Time	Time Constant
1	996.24 °C	none	1.102 s	64.46 ms
2	1037.27 °C	3.72%	1.145 s	91.64 ms
3	1038.26 °C	3.82%	1.300 s	189.6 ms
4	1091.25 °C	9.12%	1.161 s	101.75 ms
5	1309.54 °C	30.95%	1.102 s	64.46 ms

## Data Availability

Data are contained within this article.

## Abbreviations

The following abbreviations are used in this manuscript:

QNN	Quantum neural network
BPNN	Backpropagation neural network
